# Multisectoral resilience for the next global health emergency

**DOI:** 10.1136/bmjgh-2023-013320

**Published:** 2023-10-31

**Authors:** Amanda McClelland, Sulzhan Bali, Scott F Dowell, Margaret Kruk, Yee Sin Leo, Gina Samaan, William Wang, Zachary Hennenfent, Siobhan Lazenby, Anne Liu, Rhoda Kitti Wanyenze, Jennifer B Nuzzo

**Affiliations:** 1 Prevent Epidemics, Resolve to Saves Lives, New York, New York, USA; 2 Health, Nutrition, and Population, World Bank, Washington, District of Columbia, USA; 3 Vaccine Development & Surveillance, Bill & Melinda Gates Foundation, Seattle, Washington, USA; 4 Global Health and Population, Harvard University T H Chan School of Public Health, Boston, Massachusetts, USA; 5 National Public Health and Epidemiology Unit, National Centre for Infectious Diseases, Singapore; 6 WHE, World Health Organization, Geneve, Switzerland; 7 Epidemic Preparedness & Response, Gates Ventures LLC, Kirkland, Washington, USA; 8 School of Public Health, Makerere University, Kampala, Uganda; 9 Department of Epidemiology, Brown University School of Public Health, Providence, Rhode Island, USA; 10 Pandemic Center, Brown University School of Public Health, Providence, Rhode Island, USA

**Keywords:** COVID-19, Health systems, Public Health

**Summary box**

- Pandemics are more than public health emergencies; they are multisectoral crises that touch every aspect of public and private life.
- Exemplars in global health research identified six low-income and middle-income countries that acted quickly to foster coordinated multisectoral collaboration in their COVID-19 response—especially by enabling stakeholders to share and coordinate information, resources and personnel—which boosted preparedness and action and helped ensure that policy responses were equal to the magnitude of the pandemic crisis.
- Countries who have demonstrated extraordinary progress provide crucial lessons that should not be ignored, especially now as we move towards building a global health architecture that facilitates multisectoral, interdisciplinary, sustainable coordination and cooperation.

In May 2023, the WHO declared that COVID-19 is no longer a public health emergency of international concern.^1^ Simultaneously, WHO Director-General Dr. Tedros Adhanom Ghebreyesus urged countries to sustain national capacity gains and improve country readiness for future outbreaks ‘to avoid…a cycle of panic and neglect.’ As the risk of epidemics increases, health systems around the world must act preemptively to strengthen institutional resilience and preparedness for future health emergencies.

The COVID-19 pandemic was not only a public health crisis, but also a socioeconomic and educational crisis. It disrupted trade, transportation, destabilised agriculture and food systems, and undermined supply chains. It was an emergency affecting nearly every aspect of public (and private) life and demanded responses from many sectors, not just the health sector. As such, multisectoral collaboration should be incorporated as a foundational element of preparedness efforts going forward.

Researchers and policy-makers have long understood the need for multisectoral engagement. As a WHO draft report published after the 75th World Health Assembly in May 2022 put it, ‘health emergency preparedness, response and resilience (HEPR) is multisectoral by nature’.^2^ To strengthen global HEPR architecture for the future, national and international stakeholders ‘need ways of working together that deliver collaboration and coordinated, collective action.’ That means building and funding systems for emergency preparedness that work across sectors and regions—in disease surveillance, border travel and trade, risk communication, and supply chains and logistics, for example.^3^ Although the health sector has a critical role to play in health emergency prevention, preparedness and response, it cannot do it alone. A prepared and resilient health system also depends on non-health sectors, including electricity, transport and the private sector to ensure equitable access to healthcare and to minimise disruptions to essential health (and non-health) services during an emergency.^4^

Although stakeholders agree in principle that actions must be coordinated multisectorally, and some progress has been made on establishing One Health platforms, implementing multisectoral solutions remains a challenge. Yet early in the COVID-19 pandemic, policy-makers in some countries were able to put these principles into practice. They created national collaborative bodies that acted quickly during unprecedented upheaval. If we can learn from their experiences, the end of COVID-19 as a public health emergency can mark the beginning of a new phase of strengthening multisectoral systems for the future.

Through the Exemplars in Global Health research coalition, we learnt crucial, real-time lessons. Researchers from the schools of public health at Brown University, Johns Hopkins University, and Makerere University identified six low-income and middle-income countries whose pandemic response included promising practices that could be applied to other country contexts: Costa Rica, the Dominican Republic, Ghana, Sri Lanka, Thailand and Uganda.^5^ These countries demonstrated an ability to maintain essential health services (EHS) while also responding to the pandemic and controlling the spread of SARS-CoV-2, based on analysis of COVID-19 response indicators (including age-standardised death rates, cases per capita and COVID-19 test positivity rate) and health service provision indicators (including disruption to diphtheria-tetanus-pertussis immunisation rates).^6^ Selection of these countries was further validated through literature review and key informant interviews to examine in country policies and strategies implemented during COVID-19.

The selected countries shared several features that may have enabled their COVID-19 response strategies. Commonalities include strong underlying partnerships with the private sector and academic institutions, pre-existing health financing mechanisms for emergency response and EHS delivery, existing disease response capacity that could be leveraged quickly as needed, and availability/flexibility of a strong health workforce that enabled rapid mobilisation and deployment.

Among the key lessons learnt was that most of these countries acted quickly to foster coordinated multisectoral collaboration in their pandemic response, especially by enabling stakeholders across all sectors to share and coordinate information, resources and personnel. In many of the countries studied (including Costa Rica, the Dominican Republic, Sri Lanka, Thailand and Uganda), heads of state convened officials from across sectors, both public and private, and empowered them to collaborate to make policy decisions and mobilise resources for emergency response. For instance, education officials adapted learning guidelines for remote learning, finance officials mobilised emergency resources for the pandemic response, labour officials administered public assistance programmes and support for laid-off workers and other vulnerable groups, and tourism officials helped visitors navigate public health and social measures. These initiatives helped ensure that policy responses were coordinated and similar in magnitude to the pandemic crisis.

In Uganda, the Office of the Prime Minister quickly established a multisectoral, multidisciplinary National COVID-19 Task Force in March 2020. Task force members and subcommittees included officials from the Ministry of Health and other ministries—including gender, labour and social development; local government; education and sports; information and communications technology; defence, finance, planning, and economic development; and agriculture—as well as representatives from private and civil society organisations. Together, they coordinated Uganda’s pandemic preparedness activities, including contact tracing, risk communication, EHS continuity, and social and educational safety nets; building support for public health and social measures; and addressing other challenges associated with the COVID-19 pandemic, such as increases in gender-based violence.

Similarly, Costa Rica’s pandemic response was coordinated by an emergency operations centre composed of multi-institutional partners including the Ministry of Health; the Social Security Fund; the National Emergency Commission; the Ministry of National Planning and Economic Policy; the Ministry of Economy, Industry and Commerce; the Pan American Health Organization; the Joint Institute for Social Assistance; and the INCAE Business School.^7^ The country’s president also established a policy working group whose members included representatives from the ministries of health; public security; economy, industry and commerce; and tourism.^8^

Although these collaborative efforts were created specifically for the COVID-19 pandemic, some used pre-existing structures for multisectoral governance. In the Dominican Republic, the National System for Prevention, Mitigation and Response to Disasters, was established in 2002 to coordinate multisectoral, interdisciplinary response plans for many types of emergencies.^9^ Chaired by the country’s president, it developed a multiphase contingency plan for COVID-19 in March 2020, which included public health monitoring and lockdowns (with the assistance of police and security officials), community outreach and financial assistance programmes.^10^ Multisectoral data sharing among officials across ministries, such as the Ministry of Public Health and the Ministry of Defense, was key to this plan. In April of 2020, the Dominican government asked the Ministry of Defense’s Command, Control, Communications, Computers, Cybersecurity and Intelligence Centre to integrate the health system into its digital platform, enabling health officials to work with private sector partners to procure resources, view key data in real time and create predictive models for decision-making.^11^

Some countries created multisectoral governance structures quickly or used ad hoc structures. For example, in March 2020, Thailand established a Centre for COVID-19 Situation Administration (CCSA) that was chaired by the prime minister^12^ and included high-level administrators from all government ministries with the authority to implement a collaborative whole-government response. Officials submitted proposals for certain actions—including the procurement of personal protective equipment, economic support, designation of facilities for COVID-19 treatment and holiday cancellations—to the CCSA for decision, endorsement and nationwide implementation, ensuring consistency and collaboration across agencies.^13^

Some of the countries displayed commitments to multisectoral collaboration at the regional level as well. In Uganda, cross-border temperature and symptom testing made it possible for staff from the Ministry of Internal Affairs at border points of entry to intercept ill travellers before they entered the country. This collaborative effort helped control COVID-19 transmission while the country’s health facilities prepared to respond.

Across the countries studied, purposeful collaboration between health and non-health agencies and the establishment of national decision-making bodies that had formal authority, early in the COVID-19 pandemic strengthened preparedness and improved collaborative action. Now we must ask: how can these findings translate into global, regional and country policy even as COVID-19 is no longer a public health emergency? How can we build a global health architecture that facilitates multisectoral, interdisciplinary, sustainable coordination and cooperation?

Many stakeholders have already begun this work. WHO Member States have started negotiating a ‘pandemic prevention, preparedness, and response accord’ that should represent a global commitment to a concerted all-of-government, whole-of-society approach to future health emergencies.^14^ WHO has also begun the International Health Regulations amendment process and created the Preparedness and Resilience for Emerging Threats initiative, which aims to identify interdependencies between 15 critical sectors, providing actions to be taken collaboratively during the interpandemic period.^15^ Likewise, the World Bank’s Pandemic Fund can provide crucial investments that encourage multisectoral action, including a One Health approach that engages both. For multisectoral partnerships and coordination to be truly effective, they must operate with a One Health vision and proactively engage in health and non-health sectors (eg, agriculture, wildlife and environment sectors) to prevent spillovers and epidemics. In September 2023, the United Nations General Assembly High-Level Meeting on Pandemic Prevention, Preparedness and Response aims to sustain high-level political attention and prioritise the role of multisectoral preparedness and response high on the global agenda. However, establishing more overlapping institutions and initiatives will not by itself prepare us for a post-COVID-19 world.^16^

We need to empower them to act meaningfully to realise the multisectoral collaboration and coordination we seek—at the global and country level. Furthermore, planning across sectors must clarify roles and responsibilities, and be informed by lessons learnt, including about changing risk drivers and sociopolitical factors. As progress is made in discussions on global health architecture, lessons learnt in multisectoral collaboration should inform country health emergency preparedness governance and legal frameworks moving forward, coordinated by high-level leadership, to ensure sectors are actively building, testing and using systems for collaboration—well before crises emerge. We should not miss the opportunity to learn from countries that have already begun these efforts, before the next global health emergency strikes.

## Data Availability

Data available within the article or its supplementary materials.
